# Should Doctors Be Peers?

**Published:** 1896-10-31

**Authors:** 


### The Hospital

A Journal of

The Medical Sciences and Hospital Administration, with

AN EXTRA NURSING SUPPLEMENT.

Vol. XXI.—No. 527.] [Oct. 31, 1896.

### Should Doctors be Peers?

A lay contemporary, the *Evening Standard*, takes The Hospital to task for insisting that medical men of distinction should be raised to the peerage. "We imagine the family doctor," says our contemporary, "utilising any leisure moment, when tending a sick householder, to argue the political questions of the day, and solicit his patient's vote and interest with the party he favours," with the object, of course, of increasing the political influence of the profession, and so of securing its election, sooner or later, to the peerage. It is suggested that the profession will do better to "rely upon the justice of its cause, and the common sense of the public."

The public, we think, by the exercise of its common sense, has failed to grasp the situation, and in proof of this we could adduce the remarks of the contemporary we have quoted. The family doctor is the only "doctor" known to the majority of the public; and when it is suggested that a certain number of medical men should be raised to the peerage, immediately the average householder thinks of his own family physician, and, much though he respects him, would rather not be called upon to address him as "my lord" during a consultation. In this we entirely sympathise. But every doctor is not a family doctor; and this is what the public generally, and even politicians of capacity, fail to understand. Every doctor is not even a "consulting physician," or an "operating surgeon." There are among us a select few who have risen above even those ranks; and the number of such will increase as science advances and philosophic capacity becomes more marked and decisive. Of such as these, several might now be named—men whose eminence in the sciences that make up "medicine" is world-wide, and whose general culture is of the highest. Most of these men are of ripe years, and stand out by general consent conspicuous, not among the family doctors alone, but eminent and distinguished even by comparison with men of established consultant rank whether in medicine or surgery. It is of men such as these, men of the order of the present distinguished President of the British Association, that medical peers should be made.

Now, this question of peerages for doctors is a matter of some importance, first to doctors themselves, and then to the public. A peerage, in an ancient State like our own, where honours emanate from a high and venerable source, and in which they have been held in high esteem by the most honourable of our fellow-countrymen for many centuries, is not to be lightly spoken of. No doubt the hereditary peer, through long familiarity, thinks less of this distinction than the man who won his peerage but yesterday. Nevertheless, a new peerage, when honourably won and maintained with dignity, is justly considered by its holder to be the most significant and decisive of all the distinctions which his admiring contemporaries could single him out for. Of course we can well understand that people may wonder that medical men of high scientific eminence, whose fame is only second to that of the great statesman or soldier himself, should condescend to worry themselves about peerages. As a matter of fact such men do not so condescend; it is we who are of less note in the world, and who know what great and high qualities and labours have gone to the making of their scientific distinction, who "worry" for them. There can be no question that a slur is cast on medicine by those who practise it being shut out of the peerage, when lawyers, soldiers, men of commerce, poets, and painters are taken in. It will be generally admitted that medicine and surgery are, in fact, wronged in this connection. Well, we hold that they ought not to be wronged; and, at any rate, that, being wronged, they ought to be righted. Thus far we have dealt with the case entirely from the medical point of view. There is, however, a public point of view also. Whilst medical men are kept back from the first rank in social and imperial life, medical influence is maintained at a low level in every department of social and political activity. Now medical influence—that is really the influence of science, of knowledge, and of practical ideas—ought in a civilised State to be encouraged as much as possible, always and everywhere, throughout all ranks of the community. Knowledge, science, practical ideas, really constitute the most important factors in the progress, well-being, and happiness of the State. Everybody, in fact, ought to conspire to promote the acquisition and practical utilisation of such knowledge in every conceivable way. If science is to be the utiliser universally, it must first be universally honoured; and we know of no one circumstance which would give greater impetus to the progress and universal utilisation of physical and medical and sanitary science than the singling out of two or three of the most typical representatives of these sciences for elevation to the noble and distinguished ranks of the peerage.

